# Serum Cytokines and TGF-β1: A Window into Syphilis Among People Living with HIV

**DOI:** 10.3390/pathogens15030283

**Published:** 2026-03-05

**Authors:** Adriana Hernández-Pliego, Santa García-Cisneros, Dayana Nicte Vergara-Ortega, Fernando R. Esquivel-Guadarrama, Antonia Herrera-Ortíz, Cairo Toledano-Jaimes, Miguel Angel Sánchez-Alemán

**Affiliations:** 1 Escuela de Salud Pública de México, Instituto Nacional de Salud Pública, Cuernavaca 62100, Mexico; ibt.ahp@gmail.com; 2 Centro de Investigación Sobre Enfermedades Infecciosas, Instituto Nacional de Salud Pública, Cuernavaca 62100, Mexico; sgarcia@insp.mx (S.G.-C.); nicte.vergara@gmail.com (D.N.V.-O.); aherrera@insp.mx (A.H.-O.); 3 Facultad de Medicina, Universidad Autónoma del Estado de Morelos, Cuernavaca 62350, Mexico; fernando.esquivel@uaem.mx; 4 Facultad de Farmacia, Universidad Autónoma del Estado de Morelos, Cuernavaca 62210, Mexico; tjcd_ff@uaem.mx

**Keywords:** TGF beta 1, HIV, active syphilis, people living with HIV, syphilis

## Abstract

*Treponema pallidum* is the etiological cause of syphilis, and in recent years, reemergence has been reported, especially among men who have sex with men and people living with HIV (PLWH). Certain cytokines may act as hallmark biomarkers in the progression of syphilis in PLWH, and studying how the immune system works against *T. pallidum* is important, especially in PLWH, whose immune system is compromised. We evaluated the serum expressions of IFN, TNF, IL-10, TGF-β1 and IL-17 in men living with HIV (MLWH) and their association with distinct stages of syphilis. We recruited MLWH from March to October 2022. A blood sample was requested, syphilis was detected using the reverse algorithm, and antibodies were titrated to determine the stage. Each of the cytokines studied was quantified using commercial ELISA kits. The following groups were formed: active syphilis (*n* = 217), cured syphilis (*n* = 134), and without syphilis (*n* = 159). The prevalence of elevated TGF-β1 differed between groups, being highest in individuals with active syphilis (51.6%; median 319 pg/mL), followed by those with cured syphilis (41.0%; median 137.0 pg/mL). Younger participants and persons without a history of sexually transmitted infections were more likely to present with high TGF-β1 levels. TGF-β1 may act as a biomarker in active syphilis and could suppress the inflammatory response against spirochetes.

## 1. Introduction

*Treponema pallidum* subsp. *pallidum* is the etiological agent of syphilis. In 2022, the global incidence among individuals aged 15–49 years was estimated to be approximately 8 million new cases [1]. Syphilis is currently reemerging, particularly among vulnerable people such as men who have sex with men (MSM) and people living with HIV (PLWH) [2].

HIV infection is initially characterized by an increase in regulatory T cells (Tregs) that produce IL-10 and TGF-β [3,4,5]. These cytokines modulate immune activation and drive TH1 polarization, which is associated with IL-17 secretion [3,5,6]. In contrast, primary syphilis elicits a strong proinflammatory response at the chancre site and in the inguinal lymph nodes, with elevated IFN-γ and TNF levels [7]. The tertiary stage is characterized by a systemic IL-17-driven inflammatory profile [8,9,10]. During HIV–syphilis coinfection, a negative impact on CD4+ T-cell counts has been documented; however, these levels tend to recover following syphilis treatment [11]. Systemic levels of IL-10 and TNF have been reported to increase in patients with primary or secondary syphilis and subsequently decline after antibiotic therapy. Conversely, HIV viral load is positively correlated with IL-10 and TNF concentrations [12].

Given the limited data concerning immune responses to *T. pallidum* in PLWH, this study evaluated the effects of spirochetes on the profiles of CD4^+^ T-cell-associated systemic cytokines, including IFN-γ, TNF, IL-17, IL-10, and TGF-β1. Elucidating the role of these immunological biomarkers may be crucial to understanding and halting the progression of this stealth pathogen.

## 2. Materials and Methods

Between March and October 2022, men living with HIV (MLWH), attending a specialized HIV care center (CAPASITS) in Cuernavaca, Morelos, Mexico, were invited to participate in this study. Eligible participants were aged ≥18 years, were diagnosed with HIV, and provided written informed consent. Individuals who had received any antibiotic treatment within the three months preceding sample collection were excluded. Blood samples were collected in EDTA tubes (Becton Dickinson, Plymouth, UK). Sociodemographic and clinical data were obtained from the participants’ medical records. The study protocol was reviewed and approved by the Scientific Ethics Committee, the Biosafety Committee, and the Research Committee of the National Institute of Public Health of Mexico (Approval ID: 1739).

The reverse algorithm recommended by the CDC was applied to detect syphilis, using bioELISA SYPHILIS 3.0 (BIOKIT, SA-08186 Lliçà d’Amunt, Barcelona, Spain) as the treponemal test and TRUST (New Horizons Diagnostics Corporation, Columbia, MD, USA) as the quantitative nontreponemal test. Active syphilis was defined as an antibody titer ≥1:1, whereas a negative titer indicated cured syphilis [13]. For cytokine quantification, plasma samples were analyzed using the PicoKine™ quantitative ELISA kit (BOSTER Biological Technology, Pleasanton, CA, USA), with a sensitivity of 1 pg/mL. Each kit was specific for the corresponding cytokines (TGF-β1, TNF, IL-10, IL-17, and IFN-γ). The limit of quantification for all cytokines was 31.25 pg/mL; samples below this threshold were considered negative.

The population was stratified into three groups: active syphilis, cured syphilis, and no syphilis. The prevalence of each cytokine was assessed according to syphilis status, and differences were analyzed using the chi-square test. The mean concentration and 95% confidence intervals (95% CIS) of the most prevalent cytokines were calculated for each syphilis group. Differences among groups were evaluated using the Kruskal–Wallis test, followed by the Dwass–Steel–Critchlow–Fligner test. Sociodemographic and clinical information was obtained from a subsample of the population. Multivariate logistic regression analysis was performed to identify factors associated with the cytokine TGF-β1, and odds ratios and 95% CIs were reported. Statistical analyses were conducted using Jamovi 2.4.1.4 software.

## 3. Results

**Figure 1:**
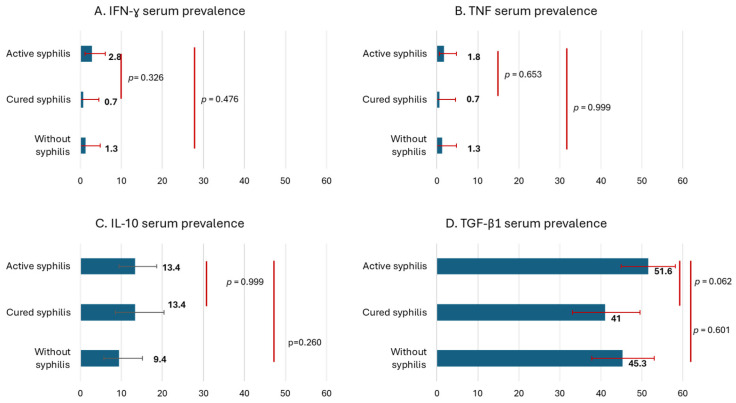
Prevalence of serum cytokines in men living with HIV, (**A**) IFN-γ, (**B**) TNF, (**C**) IL-10, (**D**) TGF-β1, stratified by syphilis infection. Bars indicate 95% confidence intervals. Comparisons were performed using the chi-square statistical test.

Blood samples were collected from 510 MLW. Of them, 217 had active syphilis, 134 had cured syphilis, and 159 had no syphilis. IFN-γ was detected in 1.8% of the participants, with a slightly higher proportion in the active syphilis group (2.8%), although this difference was not statistically significant. TNF expression levels were similar across groups, ranging from 0.7% to 1.8%. IL-10 serum detection did not significantly differ between the groups: 13.4% in participants with active syphilis and 9.4% in those without syphilis. The prevalence of elevated TGF-β1 differed marginally between the groups, with 51.6% in participants with active syphilis and 41.0% in those with cured syphilis (*p* = 0.062). All samples tested for IL-17 were negative. The cytokines analyzed are shown in Figure 1.

Interleukin-10 and TGF-β1 were detected at higher levels among MLWH and were therefore analyzed quantitatively. The mean IL-10 concentration did not significantly differ between the groups: 15.3 pg/mL in participants with active syphilis, 13.9 pg/mL in those with cured syphilis, and 8.36 pg/mL in those without syphilis. The mean concentration of TGF-β1 was 319 pg/mL in MLWH with active syphilis, 137.0 pg/mL in those with cured syphilis, and 248 pg/mL in those without syphilis; this difference was statistically significant (*p* = 0.024). TGF-β1 was the cytokine with the highest proportion among MLWH and the greatest prevalence in individuals with active syphilis; therefore, its association with demographic and clinical factors in the study population was further analyzed.

Sociodemographic and clinical information was obtained from 229 of the 510 participants who provided blood samples. When the patients were stratified by the syphilis infection status, variables such as age, education level, use of illegal drugs, time since HIV diagnosis, and viral load were not significantly different. By contrast, occupation, sexually transmitted infections (STIs), and CD4+ lymphocyte count significantly differed across the syphilis infection groups.

The differences in the frequency of TGF-β1 and odds ratios according to sociodemographic and clinical variables are presented in Table 1. Younger participants (18–29 years) and those who were unemployed had higher odds of TGF-β1 detection (OR = 1.57 and 1.72, respectively). Individuals who had ever used illegal drugs had a lower TGF-β1 proportion (32.3%). Conversely, participants without a history of STIs were more likely to present with high levels of TGF-β1 (OR = 1.38). Finally, after adjustment for the CD4+ count, HIV viral load, and other variables, MLWH with active syphilis were twice as likely to have different levels of TGF-β1 than those with cured syphilis were.

## 4. Discussion

Proinflammatory cytokines (IFN-γ and TNF) were detected at low frequencies in the study population, in contrast to regulatory cytokines (IL-10 and TGF-β1), whose prevalence was higher, as in previous reports among PLWH who are on antiretroviral therapy and have an undetectable viral load. Regulatory cytokines tend to increase as a compensatory response to a reduction in proinflammatory cytokines [14,15]. Furthermore, the levels of proinflammatory cytokines (IFN-γ and TNF) are low when HIV infection is well controlled and viral replication is suppressed [16].

IL-10 has been reported at low concentrations (4.66 and 7.05 pg/mL) in both healthy controls and PLWH with undetectable viral loads but at high concentrations (31.57 pg/mL) prior to the initiation of antiretroviral therapy (ART) when the HIV viral load is elevated [16]. In a population of MLWH where approximately 80% have an undetectable viral load, IL-10 levels would be expected to remain low. However, in our study, IL-10 was detected more frequently in groups with active and cured syphilis, and the mean IL-10 concentration was higher in both groups. These findings suggest that this regulatory cytokine is activated during HIV/syphilis coinfection, which is consistent with evidence that coinfections can modulate immune responses and increase anti-inflammatory cytokine activity.

Regulatory cytokines were detected at higher concentrations, with TGF-β specifically reported at elevated concentrations prior to antiretroviral therapy (approximately 102 pg/mL). Moreover, the expression of TGF-β is positively correlated with the HIV viral load and negatively correlated with the CD4+ count [16], suggesting that it plays an important role in modulating the immune response against HIV. In the present study, TGF-β was significantly associated with active syphilis infection, even though participants did not present the clinical symptoms of syphilis but did exhibit high antibody titers. These findings indicate a regulatory response during HIV and active syphilis coinfection. In addition, the presence of TGF-β1, a regulatory cytokine, was associated not only with active syphilis but also with a history of previous STIs.

According to a systematic review [17], the use of IL-10 and IL-17 is expected to help characterize the immunological profile of PLWH coinfected with *T. pallidum*. However, IL-17 was not detected in our study. The proinflammatory cytokines TNF and IFN-γ affect secondary lymphoid organs and trigger effector responses against coinfection. Conversely, IL-10 may suppress the IFN-γ response during active syphilis, although not completely, as it remains detectable even during cured syphilis. IL-10 is recognized as a master suppressor cytokine, yet its role in the context of *T. pallidum* infection remains unclear. Our findings align with those of Kenyon et al. [18], suggesting that IL-10 expression could represent a sustained response to HIV and a compensatory mechanism mediated by T regulatory cells to modulate the inflammatory cascade. Another potential source of IL-10 may be M2 macrophages; however, further research is needed to clarify this in the context of *T. pallidum* coinfection.

TGF-β1 has been proposed as a marker of progression from HIV infection to AIDS [19,20,21]. Babolin and colleagues reported an eightfold increase in TGF-β1 concentrations in individuals with secondary syphilis compared with healthy controls [22]. However, the role of this cytokine in *T. pallidum* infection remains unclear. To our knowledge, this is the first study to examine TGF-β1 in the context of HIV and syphilis coinfection. Our findings indicate that the systemic TGF-β1 response is predominantly associated with active syphilis, which may provide insights into the mechanisms regulating cell-to-cell communication and immune modulation during coinfection.

TGF-β1 may play a critical role in suppressing TNF-mediated or other proinflammatory responses during syphilis. This immunomodulation may lead to an increased treponemal burden, while the host remains asymptomatic and unaware of the infection. Although IL-17 has been identified as a biomarker of neurosyphilis [9], this cytokine was not detected in our study population. This absence may be attributed to optimal adherence to ART, the specific ART regimen used, and the maintenance of an undetectable viral load, resulting in a “disease-free” state. Alternatively, IL-17 may persist within secondary lymphoid organs such as the spleen or lymph nodes. Excessive concentrations of TGF-β1 may not favor the host, as they can impair nitric oxide production in macrophages and hinder B-cell antibody maturation, ultimately compromising the development of immunological memory.

Among the limitations of this study are the lack of evaluation of a broader panel of cytokines and the absence of complete clinical information for all participants. It is also important to note that the term active infection was used based on antibody titers obtained through non-treponemal tests, rather than the traditional clinical classification of syphilis into primary, secondary, and tertiary stages.

## 5. Conclusions

Serum TGF-β1 expression increases across different stages of syphilis, and this study is the first to evaluate its role in the context of HIV, identifying it as a potential biomarker for active syphilis. These findings may provide critical insights into the progression and interplay of both diseases.

## Figures and Tables

**Table 1:** Sociodemographic and clinical factors associated with serum TGF-β in men living with HIV.

	% TGF-β	cOR (95% CI)	aOR (95% CI)	*p*
Aged				
18–29 years	64.5	**1.53 (1.14–1.85)**	**1.57 (1.12–1.92)**	**0.014**
30–39 years	39.3	0.93 (0.61–1.29)	0.88 (0.54–1.28)	0.535
≥40 years	42.2	1.00	1.00	
Occupation				
Student	66.7	**1.61 (1.05–2.03)**	1.39 (0.74–1.95)	0.259
Unemployed	61.5	**1.49 (1.06–1.85)**	**1.72 (1.25–2.06)**	**0.003**
Employee	41.4	1.00	1.00	
Schooling				
Degree	47.4	1.05 (0.71–1.39)		
High School	48.5	1.07 (0.71–1.44)		
Elem/Mid school ^1^	45.3	1.0		
Illegal drugs				
Ever	32.3	**0.61 (0.48–0.85)**	**0.66(0.49–0.98)**	**0.042**
Never	52.7	1.0	1.00	
Time of HIV diagnosis				
0–1 years	59.6	**1.53 (1.09–1.89)**		
2–10 years	47.0	1.24 (0.87–1.60)		
≥11 years	37.1	1.00		
Previous STI				
Never	56.3	**1.39 (1.06–1.69)**	**1.38 (1.00–1.72)**	**0.047**
Ever	40.6	1.00	1.00	
Viral load				
Detectable	60.5	1.37 (0.99–1.70)		
Undetectable	44.1	1.00		
CD4 count				
≤499 cells/mL	54.2	**1.32 (1.01–1.63)**	1.28 (0.93–1.63)	0.122
≥500 cells/mL	41.0	1.00	1.00	
Syphilis				
Active syphilis	58.2	**1.91 (1.35–2.41)**	**2.02 (1.43–2.56)**	**<0.001**
Never syphilis	45.8	1.37 (0.92–1.87)	1.59 (0.96–2.23)	0.068
Cured syphilis	30.5	1.00		

^1^ Elementary or middle school. cOR: crude odds ratio; aOR: adjusted odds ratio. Bold. Statistical significance.

## Data Availability

The raw data supporting the conclusions of this article will be made available by the authors on request.

## Abbreviations

The following abbreviations are used in this manuscript:

PLWH	People Living With HIV
MLVH	Men Living With HIV
IFN	Interferon
TNF	Tumor Necrosis Factor
IL-10	Interleukin 10
IL-17	Interleukin 17
TGF-β1	Transforming Growth Factor Beta 1
Tregs	Regulatory T cells
